# Interaction with Virtual Humans and Effect of Emotional Expressions: Anger Matters!

**DOI:** 10.3390/jcm12041339

**Published:** 2023-02-08

**Authors:** Mariachiara Rapuano, Tina Iachini, Gennaro Ruggiero

**Affiliations:** Laboratory of Cognitive Science and Immersive Virtual Reality, CS-IVR Department of Psychology, University of Campania “Luigi Vanvitelli”, Viale Ellittico, 31, 81100 Caserta, Italy

**Keywords:** emotional virtual agents, interpersonal distance, hybrid social interactions, immersive virtual reality, anger-superiority effect

## Abstract

Today we are experiencing a hybrid real-virtual society in which the interaction with virtual humans is normal and “quasi-social”. Understanding the way we react to the interaction with virtual agents and the impact of emotions on social dynamics in the virtual world is fundamental. Therefore, in this study we investigated the implicit effect of emotional information by adopting a perceptual discrimination task. Specifically, we devised a task that explicitly required perceptual discrimination of a target while involving distance regulation in the presence of happy, neutral, or angry virtual agents. In two Immersive Virtual Reality experiments, participants were instructed to discriminate a target on the virtual agents’ t-shirts, and they had to provide the response by stopping the virtual agents (or themselves) at the distance where they could identify the target. Thus, facial expressions were completely irrelevant to the perceptual task. The results showed that the perceptual discrimination implied a longer response time when t-shirts were worn by angry rather than happy or neutral virtual agents. This suggests that angry faces interfered with the explicit perceptual task people had to perform. From a theoretical standpoint, this anger-superiority effect could reflect an ancestral fear/avoidance mechanism that prompts automatic defensive reactions and bypasses other cognitive processes.

## 1. Introduction

In everyday life, as we pursue our behavioural goals, we are used to interacting with people who may show different emotional facial expressions. Here we study the impact of emotional facial expressions on our interaction with virtual humans [1]. This issue is important if we consider that interacting with virtual humans in present times is becoming a rather common experience with regard to various technological devices. Indeed, we live in a hybrid real-virtual society where our experience with virtual humans can be described as “quasi-social” [2,3]. Sensitivity to facial expressions represents an essential component of our social life. In particular, angry facial expressions are important because they convey signals of potential threat when we interact with other people [4,5]. Another essential social component is the capacity to appropriately regulate our “private” space, that is, an emotionally tuned zone around us that cannot be intruded upon by others without causing discomfort [6,7,8,9,10].

In the proxemics literature, this zone is called “interpersonal space” and represents the optimal preferred distance from others [8,9,11]. Emotional signals strongly influence the size of interpersonal distance in such a way that it increases in uncomfortable/threatening situations and decreases in comfortable/safe situations [7,8,9,10,12,13,14]. Virtual reality technology (VR) has long been used to examine the individual’s proxemics behaviour (e.g., [15,16,17,18,19]).

Psychologists have used VR solutions with virtual humans to study individuals’ social behaviour because of their capacity to maximize ecological validity without sacrificing experimental control [15,17,20,21,22]. For example, in their influential study, Bailenson and colleagues [15] assessed individuals’ reactions to the violation of their interpersonal space by examining the interpersonal distance between virtual humans and actual participants. In Study 1, participants performed a label-reading task: they had to traverse a virtual room to approach a virtual human and read a name (on the front) or a number (on the back) on their t-shirt. In Study 2, a virtual human approached the participants who, instead, were still. The results indicated that participants treated virtual humans similarly to real humans. Indeed, they rarely violated an intimate space zone of 40 cm. This is important because, according to Hall [23], this threshold defines the private space that we only share with our intimate partners. Moreover, participants preferred a larger distance in the front compared with a back approach, and with virtual humans who engaged them in mutual gaze rather than not. This study demonstrates the effectiveness of VR in understanding how individuals interact with virtual humans, providing accurate, reliable and non-intrusive measures. Although the label-reading task did not imply a typical or engaging social interaction, it constituted a good cover story for interacting with virtual humans without disclosing the purpose of the study (i.e., an individual’s proxemics behaviour).

Other studies have confirmed the validity of VR as a tool for assessing individuals’ behaviour during dynamic social interactions (e.g., [15,17,24,25]). For example, as in the real world, people in VR can accurately estimate virtual agents’ motion to prevent collisions, anticipate their trajectory and react accordingly [25]. Moreover, people regulate interpersonal space to protect their sense of safety based on the appearance and characteristics of virtual humans (e.g., [18,19,26]). In line with this, some immersive VR studies have studied the effect of emotional facial expressions on spatial regulation mechanisms by using judgement tasks that explicitly required subjects to determine the distance at which they feel comfortable with the other’s proximity (comfort-distance) or at which they think they can reach the other with the hand (reaching-distance). The results have shown an increase of distance when interacting with virtual agents that show angry rather than happy or neutral facial expressions ([18] see also [27]). Furthermore, emotional facial expressions of virtual agents can modulate the automatic psychophysiological reactions of individuals (e.g., [19,28]). For example, Ruggiero and colleagues [19] asked the participants to interact with virtual agents exhibiting happy, angry, and neutral facial expressions. During these virtual interactions, spatial distances (comfort distance and reaching distance) and psychophysiological reactions (i.e., heart rate and skin conductance) were recorded. Results revealed that the interaction with angry rather than happy or neutral virtual agents provoked heart rate acceleration and higher levels of skin conductance, along with increased spatial distances. Therefore, we may argue that the facial expressions of virtual agents influence the behavior of individuals in a similar way to what happens in real life and that, in particular, negative threatening emotions play a key role.

However, one may argue that these findings could simply reflect a tacit compliance with the explicit instructions of choosing a distance during the interaction with the emotional virtual agents. The adoption of a task that explicitly asks for perceptual discrimination and only indirectly implies distance regulation, as in the study by Bailenson and colleagues [15], should override the criticism. Previous literature has shown that faces with fearful expressions activated the amygdala and related brain structures when subjects were asked to perform a gender decision-making task [29,30,31]. Furthermore, Frühholz and colleagues [32] used event-related potentials (ERP) to compare implicit (colour naming) and explicit (emotion judgment) tasks with emotional facial expressions and words. The results showed that the facial expressions activated early (around 100 ms post-stimulus) and late components, reflecting the enhanced encoding of emotional expressions (see also [33]). Moreover, negative faces worsened the task performance. These previous findings suggest that emotional facial expressions, by automatically capturing attentional resources, interrupt ongoing behaviours and leave fewer resources to effectively accomplish the task at hand [29,30,34,35,36,37,38].

The aim of this this study is to understand the impact of facial emotional expressions on our interaction with virtual humans by adopting a task that indirectly implied the regulation of distance. To this end, while approaching (active) and/or being approached (passive) by virtual humans wearing a t-shirt and showing happy, angry and neutral facial expressions, participants had to perform a perceptual task. More specifically, participants had to stop a virtual agent approaching them (and also stopping themselves, Experiment 2) as soon as they could identify a target-stimulus on the t-shirt. Therefore, emotional information was completely irrelevant to the explicit discrimination task. If threatening facial expressions interfere with the perceptual discrimination task, then we expect a longer response time (RT) and, as a consequence, a shorter distance when interacting with angry virtual agents than with happy or neutral ones. We also compared the active vs. passive approach to see whether participants respected the 40 cm intimate space bubble of virtual humans depending on the approach condition.

## 2. Experiment 1

In Experiment 1, the participants had to stop the approach of happy and angry virtual agents as soon as they could identify a target-stimulus (a star or a diamond) hidden among other geometric figures (circle, rectangle and triangle) on their t-shirts.

## 3. Materials and Methods

### 3.1. Participants

Thirty-two subjects (half females) aged 21–30 (M = 25.5, SD = 3) were recruited in this study in exchange for course credits. Participants were naive to the experimental hypothesis, had normal or corrected to normal vision, and did not report a history of neurological or psychiatric disease. All subjects provided written informed consent to participate in the experiment, which was approved by the Ethical Committee of the University of Campania “L. Vanvitelli” (Prot. n° 151549, n° 8, 2018), in agreement with the 2013 Helsinki Declaration [39].

### 3.2. Setting and Apparatus

The immersive virtual reality (IVR) equipment was installed in a 5 *×* 4 × 3 m room of the Laboratory of Cognitive Science and Immersive Virtual Reality (CS-IVR, Dept. Psychology). The equipment included the 3-D Vizard Virtual Reality Software Toolkit 4.10 (Worldviz, LLC, Santa Barbara, CA, USA) with the Oculus Rift DK 2 (Facebook Technologies, LLC, Menlo Park, CA, USA) head-mounted display (HMD) having two OLED displays for stereoscopic depth (resolution = 1920 × 1080; refresh rate 75 Hz). The IVR system continuously tracked and recorded the participant’s position (sample rate = 18 Hz) through a marker on the HMD. Head orientation was tracked by a three-axis orientation sensor (Sensor Bus USB Control-Unit, Santa Barbara, CA, USA). The visual information was updated in real time.

### 3.3. Virtual Stimuli and Virtual Scenario

**Figure 1:**
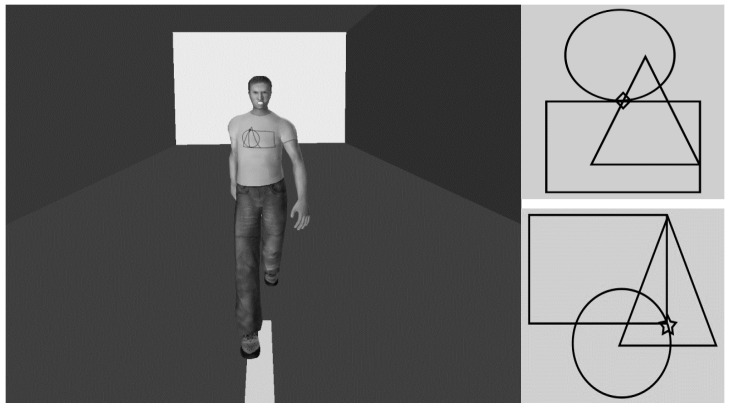
Example of virtual human and geometrical patterns with the two targets. The **left** panel shows a male virtual agent with an angry facial expression approaching participants according to their perspective; the **right** panel shows examples of a complex geometric pattern with the diamond target (**top**) and a simple geometric pattern with the star target (**bottom**).

The virtual stimuli and virtual scenario were the same as those used in a previous study [18]. We used four virtual adults (two women, two men) with happy facial expression and four virtual adults (two women, two men) with angry facial expression (tot. = 8 virtual stimuli). The height of the male virtual adults was 175 cm; and the height of the female virtual adults was 165 cm. Each virtual agent wore a light blue t-shirt showing geometrical patterns. These patterns were formed by three geometric figures (circle, rectangle, triangle) combined in such a way as to vary the degree of visual complexity (simple: the target is partially embedded within the geometrical figures; complex: the target is completely embedded within the geometrical figures). Each pattern comprised one of the two targets to be discriminated: a star or a diamond. There were 16 configurations that embedded the star and 16 configurations that embedded the diamond (Figure 1). Photoshop CS6 was used to obtain the geometric patterns on t-shirts.

### 3.4. Procedure

After giving their written consent, participants were instructed about the task, invited to wear the HMD, the Data Glove and to freely explore the virtual room. Through the HMD, participants were fully immersed in the virtual room where they could see the virtual stimuli and could make extensive exploratory movements. The Data Glove was only used during this initial training session to allow participants to perceive their arm fully stretched in the virtual scene. During this initial experience, they had to describe their feeling of presence. Next, they were led to the starting location by the experimenter and were provided with a key-press device held in their dominant hand.

The experimental session comprised two blocks, administered in a counterbalanced order. Within each block, the IVR system selected four virtual humans out of eight (two M, two F) showing happy and angry facial expressions, and 16 (eight with star and eight with diamond as target) out of 32 configurations. Each virtual agent appeared at a distance of 3 m from the participants four times (quasi-randomized order): twice wearing the t-shirt with the simple/complex configuration and twice wearing the t-shirt with the star/diamond as target, resulting in 16 trials per block (tot. = 32 trials for both blocks). A 5 min break was introduced between the two blocks with the HMD taken off.

At the beginning of the experimental session, to familiarize themselves with the procedure, participants received a four-trial training session with virtual agents not included in the task. Participants were instructed to press the key device as soon as they could discriminate a star or diamond on the virtual agent’s t-shirt and to name the target. Participants performed the task by standing still while the virtual agents walked toward them (speed = 0.5 ms^−1^) until they stopped them by a button press when they could discriminate the target. The experimental flow included: task instructions (5 s), fixation cross (300 ms), virtual agent, and participants’ response. After each block, the experimenter checked whether the participants performed the task correctly. All participants correctly identified the target. The entire experimental procedure lasted about 15 min.

## 4. Results

### 4.1. Data Analysis

In each trial, the participant-virtual agent distance (cm) and Response Time (RT, s) were recorded. The mean distances and the mean RTs were computed for each condition and then analysed through two separate ANOVAs. A 2 × 2 × 2 ANOVA for repeated measures was used, with “Facial expression” (Happy-Angry), “Target” (Star-Diamond) and “Geometrical pattern” (Simple-Complex) as within factors. All values 2.5 ± SDs were discarded (i.e., 0.9% of the dataset). The Tukey’s HSD post-hoc test was used to analyse the significant effects. The magnitude of significant effects was expressed by partial eta-squared (η*^2^_p_*). Finally, a Pearson correlation analysis was performed on mean distances and mean RTs as a function of the two emotions.

### 4.2. Response Time

The ANOVA on RTs showed a main effect of Facial expression (F(1, 31) = 11.12, *p* = 0.002, η*^2^_p_* = 0.26). RTs were longer when interacting with Angry (M = 3.61 s, SD = 0.95) than Happy (M = 3.43 s, SD = 1.05) virtual agents. A main effect of Target also emerged (F(1, 31) = 129.16, *p* = 0.0001, η*^2^_p_* = 0.81), with participants who were slower to respond when they had to identify the Diamond (M = 3.98 s, SD = 0.87) than the Star (M = 3.06 s, SD = 0.91). The analysis also showed a main effect of Geometrical pattern (F(1, 31) = 17.41, *p* = 0.0002, η*^2^_p_* = 0.36). Not surprisingly, RTs were longer when the virtual agents wore a t-shirt with complex (M = 3.61 s, SD = 0.96) as opposed to simple patterns (M = 3.43 s, SD = 1.04).

Facial expression and Target significantly interacted (F(1, 31) = 22.16, *p* = 0.00005, η*^2^_p_* = 0.42). The post-hoc analysis showed that RTs to discriminate the target Star combined with Happy facial expressions were shorter than all other combinations (at least *p* < 0.001). Instead, with the target Diamond there was no significant difference between Angry and Happy facial expressions (*p* = 0.93). Finally, RTs were shorter with Angry facial expressions combined with the target Star than with the target Diamond (*p* = 0.0002). Facial expression and Geometrical pattern significantly interacted (F(1, 31) = 5.29, *p* = 0.03, η*^2^_p_* = 0.14). The effect was due to the Happy facial expression combined with the Simple Geometrical pattern that required shorter RTs than all other conditions (at least *p* < 0.001). No other significant comparison emerged.

**Figure 2:**
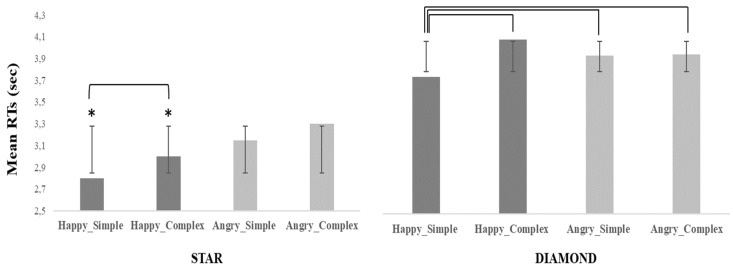
Three-way Interaction on mean RTs (Experiment 1). The graph shows the mean Response Times (in s) as a function of the two Facial expressions (Happy-Angry), the kind of Target (Star-Diamond) and the kind of Geometrical pattern (Simple-Complex). The asterisk indicates the experimental condition(s) that differs significantly from all others. Parentheses indicate experimental conditions that differ significantly from each other. Error bars represent the standard deviation.

Finally, a three-way interaction appeared (F(1, 31) = 8.23, *p* = 0.0007, η^2^*_p_* = 0.21) (Figure 2). The effect was due to the fact that RTs were shorter than all other conditions (at least *p* = 0.007) with the target Star when interacting with Happy virtual agents and both Simple and Complex patterns. As regards the target Diamond, RTs were shorter with Happy virtual agents and the Simple pattern than with other conditions (at least *p* = 0.01). As regards the target Star, in the presence of Happy agents, RTs were shorter with the Simple than Complex pattern (*p* = 0.007). These results suggest that the effect of emotional expressions was affected by the difficulty of the perceptual discrimination induced by the complexity of materials.

### 4.3. Distance

A main effect of Facial expression appeared (F(1, 31) = 40.58, *p* = 0.000001, η*^2^_p_* = 0.57). The distance was shorter when participants interacted with Angry (M = 120.10 cm, SD = 47.83) than Happy (M = 130.55 cm, SD = 52.33) virtual agents. A main effect of Target was observed (F(1, 31) = 190.28, *p* = 0.000001, η*^2^_p_* = 0.60). The distance was shorter when participants had to identify the target Diamond (M = 101.64 cm, SD = 43.63) than the target Star (M = 101.64 cm, SD = 43.63).

Moreover, the analysis showed a main effect of Geometrical pattern (F(1, 31) = 12.86, *p* = 0.001, η*^2^_p_* = 0.29). The distance was shorter when the virtual agents wore a t-shirt with a Complex (M = 121.18 cm, SD = 49) than a Simple (M = 129.47 cm, SD = 51.43) geometrical pattern.

Facial expression and Target significantly interacted (F(1, 31) = 8.30, *p* = 0.007, η*^2^_p_* = 0.21). The post-hoc test revealed that when the Happy facial expression was combined with the target Star, the distance was larger than for all other conditions (at least *p* = 0.0002). In regard to the target Diamond, the distance was larger with Happy than Angry virtual agents (*p* = 0.0002). Finally, the distance was larger with the Star than the Diamond in the presence of Angry virtual agents (*p* = 0.0002).

**Figure 3:**
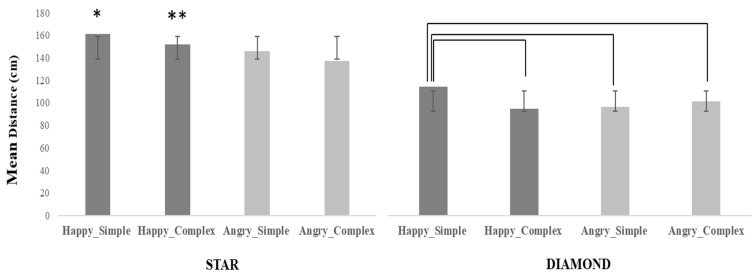
The graph shows the mean distance (in cm) as a function of the two Facial expressions (Happy-Angry), the kind of Target (Star-Diamond), and the kind of Geometrical pattern (Simple-Complex). The asterisk indicates the experimental condition(s) that differs significantly from all others (except the combination Happy/Star/Complex). The double asterisk indicates that the combination Happy/Star/Complex significantly differs from all Diamond conditions. Parentheses indicate experimental conditions that differ significantly from each other. Error bars represent the standard deviation.

Furthermore, a significant interaction between Facial expression and Geometrical pattern appeared (F(1, 31) = 10.72, *p* = 0.003, η*^2^_p_* = 0.26). The effect was due to the combination of a Happy facial expression and a Simple Geometrical pattern with a larger distance than all other conditions (at least *p* = 0.0002). Finally, a three-way interaction was observed (F(1,31) = 11.12, *p* = 0.002, η*^2^_p_* = 0.26) (see Figure 3). The post-hoc test revealed that the distance was larger than other conditions (at least *p* = 0.002) with the combination Happy/Star/Simple, except the combination Happy/Star/Complex (*p* = 0.16). In the latter, the distance was larger than all Diamond conditions (at least *p* = 0.002). As regards the target Diamond, distance was larger with Happy faces and Simple patterns than other conditions (at least *p* = 0.013). This suggests that the effect of emotional expressions was affected by the difficulty of the perceptual discrimination induced by the materials.

### 4.4. Correlation Analysis

A Pearson correlation analysis showed that distance and RT were negatively related to each other. Therefore, the shorter the distance between the participant and the virtual agents exhibiting any facial expression, the longer the RTs (at least r = −0.93, *p* < 0.0001). This correlation confirmed that distance and time were related to each other in the sense that the more time participants needed to discriminate the target, the more they allowed the virtual agent to get closer.

## 5. Discussion

The results showed that participants had more difficulty when dealing with angry than happy virtual agents, even though facial expressions were irrelevant to the task of identifying a target on t-shirts. This difficulty was reflected in a longer processing time and a shorter distance between the participant and the virtual agent. Instead, when interacting with happy virtual agents, the participants needed a shorter time and stopped them at larger distances. However, the results showed that the effect of emotional expressions was influenced by the difficulty of perceptual discrimination induced by the materials. Moreover, only a passive approach was used in this experiment. We know that an active approach can exert a specific influence on the regulation of the space around the body, and this is important to understanding whether people tend to respect the intimate space of virtual agents or not (e.g., [15,16,18]. Finally, there was no control condition for emotional stimuli (i.e., a neutral agent).

## 6. Experiment 2

In this experiment, we added an active approach condition and a neutral facial expression. Moreover, to keep the effect of the materials under control, we reduced the complexity of the geometric patterns and chose two targets of similar difficulty: a diamond and a rectangle. We expected to replicate the effect of emotion: a longer RT and a shorter distance in the presence of angry expressions compared to other facial expressions. This effect should be similar in the passive and active approach conditions.

## 7. Materials and Methods

### 7.1. Participants

Forty subjects (21 females) aged 20–30 (M = 23.37, SD = 3.10) were recruited in exchange for course credits. Participants were naive to the experimental hypothesis, had normal or corrected to normal vision, and they did not report a history of neurological or psychiatric disease. All subjects provided written informed consent to participate in the study. The study was approved by the Ethical Committee of the University of Campania “L. Vanvitelli” (Prot. n° 151549, n° 8, 2018), in agreement with the 2013 Helsinki Declaration [39].

### 7.2. Setting and Apparatus

The experimental setting and the virtual equipment were the same as those of the previous experiment.

### 7.3. Virtual Stimuli and Virtual Scenario

**Figure 4:**
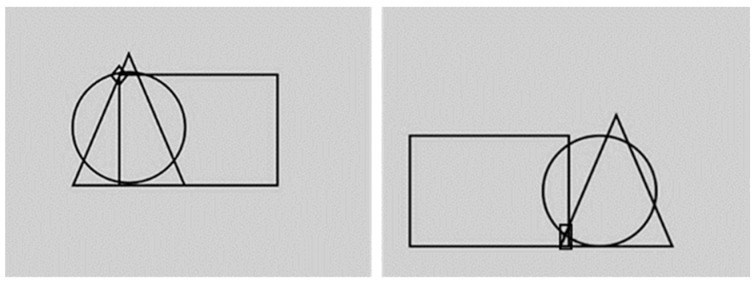
Examples of geometrical patterns. The figure shows two different geometrical patterns with the target diamond (**left**) and the target rectangle (**right**).

The virtual stimuli (except for the models on the t-shirts) and the virtual scenario were the same as those used in the previous experiment. In addition, we also selected neutral male and female virtual agents. Therefore, the sample comprised: two virtual males and two virtual females with happy facial expressions, two virtual males and two virtual females with angry facial expressions, and two virtual males and two virtual females with neutral facial expressions (tot. = 12 virtual stimuli). Each virtual agent wore a light blue t-shirt with a geometric pattern consisting of three simple figures (circle, rectangle, and triangle). One of the two targets, either a diamond or rectangle, could appear in different positions within the geometric pattern (Figure 4).

### 7.4. Procedure

The procedure was the same as Experiment 1 except that here we added the active approach condition. Thus, participants were asked to walk towards the happy, angry and neutral virtual agents and to stop as soon as they could discriminate the target on their t-shirt (active approach) or to stop the virtual agents walking towards them as soon as they could discriminate the target (passive approach). The experiment comprised two blocks, one for the active approach and one for the passive approach, administered in a counterbalanced order. Within each block, the IVR system selected six virtual agents (three females) showing happy, angry and neutral facial expressions. Each virtual agent appeared four times (quasi-randomized order), twice with the target diamond and twice with the target rectangle, resulting in 24 trials per block (tot. = 48 trials). Therefore, within each block, half of the times the target was represented by the diamond and the other half by the rectangle. A 5 min break was introduced between the two blocks with the HMD taken off. After each block, the experimenter checked whether the participants performed the task correctly. All participants correctly identified the target. The entire experimental procedure lasted about 20 min.

## 8. Results

### 8.1. Data Analysis

The mean participant-virtual agent distances (cm) and the mean RTs (s) for each facial expression within each approach were calculated. A 2 × 3 repeated-measure ANOVA with “Approach” (Active-Passive) and “Facial expression” (Happy-Angry-Neutral) as within factors was used to analyse the mean distances and RTs. All values 2.5 ± SDs were discarded (i.e., 0.2% of the dataset). A Tukey’s HSD post-hoc test was used to analyse the significant effects. The magnitude of significant effects was expressed by partial eta-squared (η*^2^_p_*).

Finally, a Pearson correlation analysis was performed on mean distances and RTs as a function of Approach and Facial expressions.

### 8.2. Response Time

The ANOVA showed a significant main effect of Approach (F(1, 39) = 113.55, *p* = 0.000001, η*^2^_p_* = 0.74). RTs were longer in the Active (M = 3.97 s, SD = 1.28) than Passive (M = 1.95 s, SD = 0.55) approach. A main effect of Facial expression appeared (F(2, 78) = 9.53, *p* = 0.0002, η*^2^_p_* = 0.20). The post hoc test showed that RTs were longer when interacting with Angry (M = 3.15 s, SD = 1.5) than Happy (M = 2.80 s, SD = 1.30) and Neutral virtual agents (M = 2.94 s, SD = 1.41) (at least *p* = 0.03). There was no significant difference between Happy and Neutral virtual agents (*p* = 0.18). Finally, no significant interaction emerged (F(2, 78) = 2.88, *p* = 0.06).

### 8.3. Distance

A main effect of Approach emerged (F(1, 39) = 23.59, *p* = 0.00002, η*^2^_p_* = 0.38). The distance was larger in the Passive (M = 200.90 cm, SD = 27.53) than Active approach (M = 165.26 cm, SD = 49.08). A main effect of Facial expression was observed (F(2, 78) = 13.02, *p* = 0.000013, η*^2^_p_* = 0.25). The post-hoc test showed that the distance was shorter when participants interacted with Angry (M = 174.32 cm, SD = 43.30) than Neutral (M = 185.19 cm, SD = 43.67) and Happy (M = 189.77 cm, SD = 42.74) virtual agents (at least *p* = 0.002). There was no significant difference between Happy and Neutral virtual agents (*p* = 0.31).

**Figure 5:**
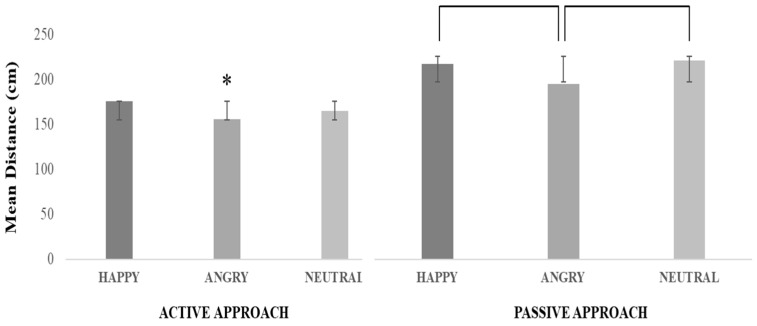
Interaction Approach and Facial expression (Experiment 2). The graph shows the mean distance (in cm) as a function of the Approach condition (Active-Passive) and Facial expressions (Happy-Angry-Neutral). The asterisk indicates the experimental condition(s) that differs significantly from all others (except the combination of Active/Neutral virtual agents). Parentheses indicate experimental conditions that differ significantly from each other. Error bars represent the standard deviation.

Approach and Facial expression significantly interacted (F(2, 78) = 3.36, *p* = 0.04, η*^2^_p_* = 0.08) (Figure 5). The Tukey’s post-hoc test revealed that the distance was shorter in the Active approach with Angry virtual agents than in all other conditions (at least *p* = 0.01) except one: the Active approach with Neutral virtual agents (*p* = 0.13). Moreover, in the Active approach the distance was shorter with Neutral than Happy virtual agents (*p* = 0.04). Regarding the Passive approach, the distance was shorter with Angry than Happy and Neutral virtual agents (at least *p* = 0.05). There was no significant difference between Happy and Neutral virtual agents (*p* = 0.99).

### 8.4. Correlation Analysis

A Pearson correlation analysis showed that distance and RT were negatively related to each other, as shown in Table 1. In both Active and Passive approaches, the shorter the distance between the participants and the virtual agents with any facial expression, the longer the RTs (at least r = −0.33 and *p* < 0.05).

## 9. Discussion

The results of Experiment 2 confirmed the previous ones, showing that virtual agents with angry facial expressions compared to neutral and happy ones caused a lengthening of processing time and a reduction of distance. These effects appeared in both passive and active approach conditions, although distance was overall larger and time faster in the former. Moreover, participants never violated the distance of 40 cm. It is important to note that the results were obtained after reducing the complexity of the geometric configurations to be identified and after adding a neutral stimulus as a control.

## 10. General Discussion

In the current study, we investigated the effect of threatening emotional stimuli on a perceptual discrimination task as an indirect index of interpersonal distance regulation between real individuals and virtual humans. To this end, participants were explicitly instructed to discriminate a target on the virtual agents’ t-shirts and to stop the virtual agents (or themselves) at the distance where they could identify the target. These virtual agents could show happy, angry or neutral (Experiment 2) facial expressions. Facial expressions were completely irrelevant to the discrimination task, and distance regulation was indirectly implied [15]. To avoid a possible confounding effect, participants were told in advance that the perceptual discrimination task involved an interaction with virtual humans. Specifically, they were informed that the target to be identified would be on the t-shirt worn by virtual humans.

The results of Experiments 1 and 2 showed that the perceptual discrimination implied a longer response time and a shorter distance when t-shirts were worn by angry rather than happy or neutral virtual agents. One possible interpretation of this finding is that angry faces interfered with the perceptual discrimination task by attracting processing resources and thus lengthening the performance. This is consistent with evidence suggesting that the need for defense requires the ability to interrupt ongoing activities and automatically reallocate cognitive resources to potentially threatening events [38,40,41,42,43].

Proxemics literature has also shown an effect of threatening stimuli on distance regulation, but an increase of distance is usually reported (e.g., [6,12,18,19,44]). However, we must consider that proxemics literature, regardless of the methodology used (observational, laboratory, projective, and so on; [45]), requires participants to determine their comfort distance from other people and not to engage them in another cognitive task. Instead, our experimental paradigm requires a perceptual discrimination task while threatening stimuli urge available resources [35,36,46]. Consistently, all participants accomplished their perceptual task accurately, but the presence of angry virtual agents determined a longer response time and a shorter interpersonal distance. In this regard, several studies have provided evidence of increased attention capture by threatening faces (e.g., [47,48,49]. This so-called anger superiority effect is consistent with the evolutionary role of the fear/avoidance system, which requires rapid encoding of threatening stimuli to elicit defensive responses at the expense of other ongoing tasks [42,50,51,52,53,54].

The effect of angry facial expressions has a bearing on the nature of the hybrid relationship between real and virtual humans. Indeed, even though people know they are immersed in an artificial world, they cannot avoid reacting as if they were facing real people: they treat virtual agents similarly to real humans (e.g., [55,56]). This conclusion is reinforced by another finding: people never violated the distance of 40 cm from the virtual agents, in both passive and active approach conditions [15]. In other words, it appears that people represent the minds of virtual agents in a similar way as they do in reality, that is, by attributing “human” intentionality to them [57]. From this perspective, we can understand why technological relationships can be defined as “quasi-social” [2]. From an applied perspective, this suggests that virtual agents can be exploited to make virtual programs, for example those aimed at rehabilitation or cognitive potentiation, more engaging and natural.

## 11. Future Studies

To the best of our knowledge, this is the first work that attempts to understand the implicit influence of facial expressions on interpersonal distance regulation using a perceptual discrimination task. Additional studies are needed to further distinguish the indirect effect of negative emotions on task performance and spatial regulation. For example, the participant-virtual human starting distance could be varied (e.g., 3 m, 1 m, or 60 cm). This would allow us to assess participants’ reaction to a possible violation of their intimate space and better understand the mechanisms of implicit spatial regulation in virtual contexts.

## Figures and Tables

**Table 1:** Correlation Between the Mean Distance and the Mean Response Time as a Function of the Approach and Facial expression Conditions (N = 40).

	Active Happy	Active Angry	Active Neutral	Passive Happy	Passive Angry	Passive Neutral
Active Happy-RT	−0.6737 ^	−0.3952 ^§^	−0.4008 ^§^	−0.2819	−0.3386 ^§^	−0.2281
Active Angry-RT	−0.4301 *	−0.4696 *	−0.3319 ^§^	−0.1986	−0.2052	−0.1716
Active Neutral-RT	−0.5767 ^	−0.4264 *	−0.5170 *	−0.2802	−0.2760	−0.2799
Passive Happy-RT	−0.2580	−0.1151	0.0943	−0.7958 ^	−0.5896 ^	−0.6515 ^
Passive Angry-RT	−0.0557	0.0210	0.0612	−0.4735 *	−0.8185 ^	−0.7134 ^
Passive Neutral-RT	−0.1418	−0.0085	−0.1302	−0.7581 ^	−0.7634 ^	−0.9691 ^

Pearson’s R*s* are reported in the table. Significant effects are indicated as follows: ^§^ *p* < 0.05; * *p* < 0.01; ^ *p* < 0.0001.

## Data Availability

The data presented in this study are available on request from the corresponding author.
